# Compact Surface Plasmon Resonance IgG Sensor Based on H-Shaped Optical Fiber

**DOI:** 10.3390/bios12030141

**Published:** 2022-02-25

**Authors:** Yijian Huang, Ying Wang, Gaixia Xu, Xing Rao, Jiaxiong Zhang, Xun Wu, Changrui Liao, Yiping Wang

**Affiliations:** 1 Key Laboratory of Optoelectronic Devices and Systems of Ministry of Education, College of Physics and Optoelectronic Engineering, Shenzhen University, Shenzhen 518060, China; huangyijian@email.szu.edu.cn (Y.H.); 2060453012@email.szu.edu.cn (X.R.); 2070456076@email.szu.edu.cn (J.Z.); 2100453060@email.szu.edu.cn (X.W.); cliao@szu.edu.cn (C.L.); ypwang@szu.edu.cn (Y.W.); 2 Guangdong Key Laboratory for Biomedical Measurements and Ultrasound Imaging, School of Biomedical Engineering, Shenzhen University Health Science Center, Shenzhen 518060, China; xugaixia@szu.edu.cn; 3 Shenzhen Key Laboratory of Photonic Devices and Sensing Systems for Internet of Things, Guangdong and Hong Kong Joint Research Centre for Optical Fibre Sensors, Shenzhen University, Shenzhen 518060, China

**Keywords:** surface plasmon resonance, biosensor, polarization-maintaining fiber, chemical corrosion

## Abstract

A compact surface plasmon resonance sensor based on an H-shaped optical fiber is proposed and demonstrated. The H-shaped optical fiber was fabricated experimentally by using hydrofluoric acid to controllably corrode the polarization-maintaining fiber. A satisfactory distance between the outer surface of the fiber and the core can be achieved, and then the surface plasmon resonance effect can be excited by coating a metal film of appropriate thickness on the surface of the fiber. This technology can realize the preparation of multiple samples at one time, compared to the traditional side-polishing technique. The H-shaped optical fiber obtained from corrosion exhibits a high surface quality and short lengths, down to only a few hundred microns. The effects of the proposed H-shaped optical fiber on spectral properties are induced by process parameters, including fiber remaining thickness, coating thickness and fiber length, and were investigated in detail. The prepared sensor was used for the specific detection of human IgG, and the minimum human IgG concentration that the sensor can distinguish is 3.4 μg/mL. Such a compact surface plasmon resonance fiber sensor has the advantages of an easy fabrication, good consistency and low cost, and is expected to be applied in the specific detection of biomarkers.

## 1. Introduction

In recent years, surface plasmon resonance (SPR) technology has been extensively investigated. It has gradually been used in practical applications in biomedicine, chemistry, environment, food safety, and other fields [1,2,3], because SPR technology has the advantage of a high sensitivity to the refractive index of external environmental media. In SPR-based optical sensing technology, the dynamic processes of bio-molecular interactions can be identified by detecting slight changes in the refractive index. This method is convenient for detection because it does not require radioactive or fluorescent labeling of the biological samples to be tested [4,5]. In addition, SPR sensors also have the characteristics of a fast response, real-time monitoring, high accuracy, and easy fabrication. At present, SPR sensors are divided according to their structures, mainly including prism coupling [6,7], integrated optical waveguide coupling [8], grating coupling [9] and optical fiber coupling [10]. Among these sensors, optical fiber SPR sensors with specific structures have the advantages of a small size, low cost, and multi-channel cascades and can be applied to multi-field online remote monitoring. For example, they may be used for in situ online real-time monitoring in the field of biochemistry and are suitable for harsh environments, such as those with a high temperature, high pressure and narrow design space. Therefore, optical fiber SPR sensors have more potential in sensing and detection than other SPR sensing structures.

The incident light is limited in the fiber core due to total reflection at the interface between the fiber core and the cladding, thus preventing the light from contacting the surrounding medium. In order to excite SPR on the surface of the optical fiber, the optical fiber’s structure needs to be changed so that the light transmitted in the core can leak to the surface of the optical fiber. In 1993, Jorgenson et al. excited SPR on an optical fiber by directly removing the optical fiber cladding and depositing metal on the fiber core [11]. After that, many fiber structures that can be used to excite SPR have been proposed, such as a hetero-core structure [12], D-type fiber [13,14,15,16], tapered fiber [17,18,19], U-shaped fiber [20], fiber grating [21,22] and photonic crystal fiber [23,24,25]. Among them, SPR excited by a D-type fiber has been studied previously and applied more widely. This kind of optical fiber SPR sensor has a high sensitivity and resolution, but the manufacturing process is complex and time consuming.

In this paper, a new fabrication method of fiber SPR sensors is proposed. Hydrofluoric acid was used to corrode the polarization-maintaining fiber (PMF). Due to the difference in the corrosion rate between the stress section in the PMF and other parts, after the corrosion was completed, the surface of the PMF in the original stress section direction was closer to the fiber core, while more cladding areas were retained in the other direction; finally, the corroded PMF became an H-shaped optical fiber. The minimum distance between the corrosion surface and the optical fiber core can be controlled below 3 μm by an appropriate corrosion time so that H-shaped optical fiber can be used to excite SPR after metal film coating. In this paper, the preparation process and spectral characteristics of the H-shaped optical fiber are studied in detail. Through the surface modification of the H-shaped optical fiber, we also use the prepared compact sensor to implement the specific detection of human IgG.

## 2. Materials and Methods

### 2.1. Materials

The 1-ethyl-3-(3-(dimethylamino)propyl)-carbodiimide hydrochloride (EDC), hydrofluoric acid (HF, 40% aqueous solution), and N-hydroxysulfosuccinimide (NHS) were purchased from Shanghai Aladdin Biochemical Technology Co., Ltd. (Shanghai, China) 11-mercaptoundecanoic acid (MUA) was purchased from Shanghai yuanye Bio-Technology Co., Ltd. (Shanghai, China) Bovine serum albumin (BSA, pH 7), phosphate-buffered saline (PBS, pH 7.4), and sodium hydroxide (NaOH) were purchased from Sigma-Aldrich (St. Louis, MO, USA). Human IgG and goat anti-human IgG were purchased from Proteintech Group Inc. (Wuhan, China). Rabbit IgG was obtained from Bioworld Technology CO., Ltd. (Nanjing, China). All these reagents were used without further purification. EDC/NHS solution and NaOH solution were prepared with PBS buffer. The PMF (PM1550_125-13/250, YOFC) utilized for the experiment was purchased from Yangtze Optical Fibre and cable Co., Ltd. (Wuhan, China).

### 2.2. Fabrication of the H-Shaped Optical Fiber

**Figure 1:**
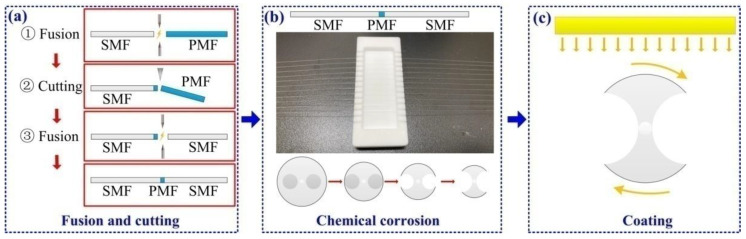
Schematic diagram of the H-shaped optical fiber preparation and coating process. (**a**) Fusion and cutting. (**b**) Chemical corrosion. (**c**) Vacuum magnetron-sputtering coating.

Figure 1 shows the manufacturing process of the H-shaped optical fiber. First, we fused the PMF with the single-mode fiber (SMF), then accurately cut the PMF under the optical amplification system [26] to ensure that the PMF of a specific length remained connected to the SMF, and finally connected another SMF to the PMF, as shown in Figure 1a. The cladding alignment mode was used in the fusion process to ensure that the fiber fusion could be carried out unimpeded. After the optical fiber fusion, these fibers were placed in a customized groove and we ensured that the PMF was in the middle of the groove, as shown in Figure 1b. After fixing the optical fiber, hydrofluoric acid with 40% concentration could be dropped into the groove to start the fiber corrosion. Figure 1b also shows the change process of the PMF end face during corrosion, finally becoming the H-shaped optical fiber. Figure 1c shows the schematic diagram of the H-shaped optical fiber coating process after corrosion. In the experiment, the rotation function of the optical fiber sample table of the magnetron-sputtering coating instrument was used to rotate the optical fiber along the optical axis, so that the surface of the H-shaped optical fiber could be evenly coated with metal film. During coating, the current and air pressure of the coating instrument were set to 40 mA and 2.0 Pa, respectively. Under this setting, the coating time increased by one minute, and the thickness of gold film on the surface of optical fiber increased by about 3 nm.

**Figure 2:**
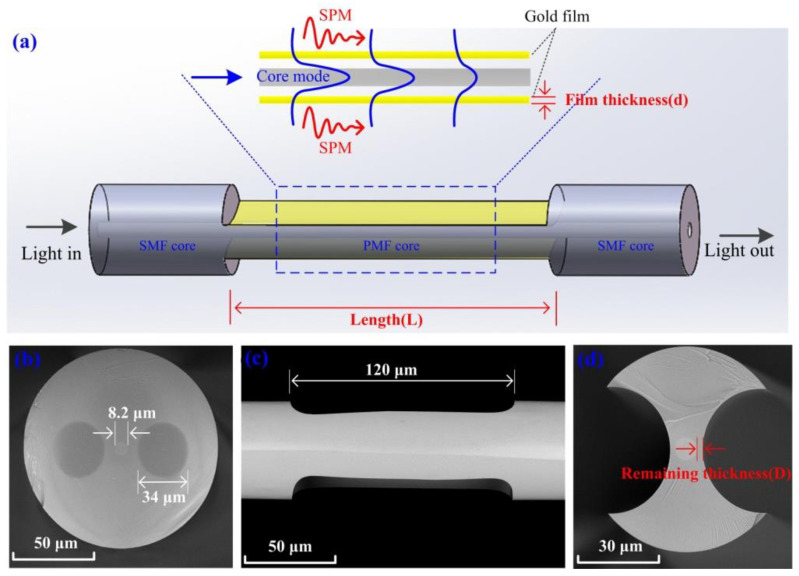
(**a**) Schematic diagram of sensing structure. (**b**) End view of PMF. (**c**) Side view of H-shaped optical fiber. (**d**) End view of H-shaped optical fiber.

Figure 2a shows the structural diagram of the sensor, including the input SMF, the H-shaped optical fiber of a small length (L) and the output SMF. The surface of the H-shaped optical fiber was coated with a gold film of appropriate thickness (d). When incident light is coupled from the input SMF into the core of the H-shaped optical fiber and transmitted forward, because the corroded surface at the original stress section direction is close enough to the H-shaped optical fiber core, light will leak from the core and form an evanescent field near the surface. When the propagation constants between the optical fiber core mode and the surface plasmon mode are equal or similar, the SPR effect near the gold layer will be excited. Figure 2b shows the end face structure of the PMF used in the experiment before corrosion. The core diameter of the PMF is equal to the diameter of the SMF core, which is 8.2 μm. The stress section diameter of the PMF is 34 μm. Figure 2c,d shows the side and end faces of the H-shaped optical fiber, respectively. It can be seen that remaining thickness (D) as well as the distance between the fiber surface and the fiber core in the direction of the original stress section is only a few microns. Although the corrosion degree of the optical fiber cladding is directly proportional to the corrosion time under a certain hydrofluoric acid concentration [27], due to the limitations of optical fiber manufacturing technology, there will be slight differences in the transverse geometric dimensions of optical fibers at different axial positions. Thus, it is difficult to precisely control the distance from the surface to the core of the H-shaped optical fiber only by adjusting the corrosion time. In the experiment, real-time monitoring of optical fiber transmission loss was mainly used to control the corrosion process of the optical fiber. When the transmission loss reaches about 1.2 dB at 1550 nm, the corroded fiber surface will be close to the optical fiber core, with a remaining thickness of 0 μm.

## 3. Results

**Figure 3:**
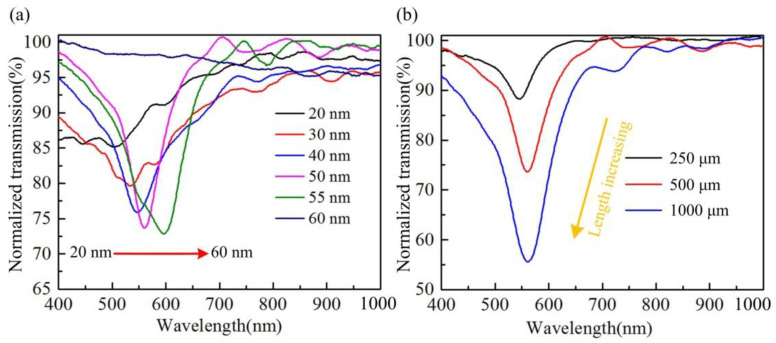
(**a**) Transmission spectra of H-shaped optical fiber with different gold film thicknesses. The length and remaining thickness of these fibers are consistent, at 500 μm and 0 μm, respectively. (**b**) Transmission spectra of H-shaped optical fiber with different lengths. The gold film thickness and remaining thickness of these fibers are consistent, at 50 nm and 0 μm, respectively.

SPR sensors usually have an optimal metal film thickness to optimize the spectral shape of the resonance peak. In order to obtain the optimal film thickness, the lengths of all H-shaped optical fibers are fixed at 500 μm and the remaining thickness is 0 μm. The optimal gold film thickness for exciting SPR is explored by coating gold films of different thickness on the surface of the optical fiber and testing the spectrum of the fiber. When the sputtering current, chamber pressure and other parameters remain constant, gold films with different thicknesses can be coated on the fiber surface by changing the coating time. The thicknesses of gold film on the surface of the optical fiber are 20, 30, 40, 50, 55 and 60 nm, respectively (coating times: 6.7, 10, 13.3, 16.7, 18.3 and 20 min, respectively). The H-shaped optical fiber spectra in Figure 3a show that when the thickness of the gold film is 50 nm, the resonant peak of the fiber has the best extinction ratio and full width at half maximum.

The traditional side-polished optical fiber is limited by machining. There are transition and uniform zones, and the length of the uniform zone is difficult to control below 1 mm. Therefore, the limitations of fiber length make these sensors unusable in some specific environments. At the same time, few papers have reported SPR optical fiber sensors below 1 mm. In order to implement a compact fiber SPR sensor, we study the effect of H-shaped optical fiber length on the transmission spectrum. In the experiment, three fibers with lengths of 250 μm, 500 μm and 1000 μm were prepared. The remaining thickness and gold film thickness of the fibers were 0 μm and 50 μm, respectively. This ensures that there is a strong enough evanescent field that can spread to the surface of the gold film and medium. The transmission spectra of the three fibers are shown in Figure 3b. They show that the resonance peak of the H-shaped optical fiber with a longer length is deeper, because as the length increases, more core-mode light will leak onto the gold film surface to excite a stronger SPR.

**Figure 4:**
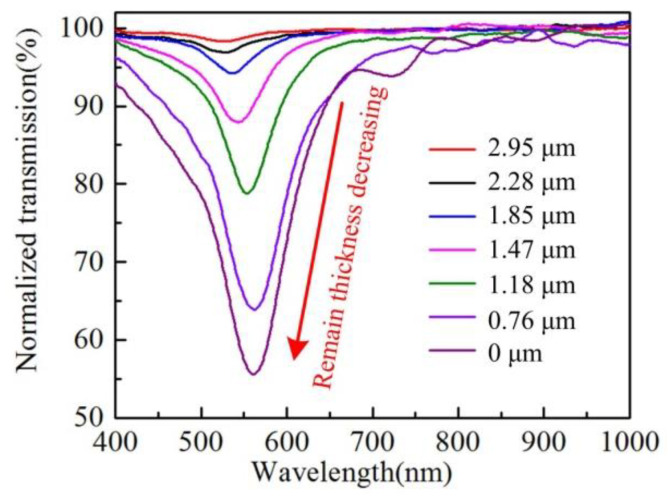
Transmission spectra of H-shaped optical fiber with different remaining thickness. The length of the corroded PMF contained in these fibers is 1000 μm.

With the decrease in the remaining thickness, the evanescent field near the gold film surface increases gradually, which will lead to strong SPR excitation. It can be inferred that the transmission resonance peak of the H-shaped optical fiber will be more obvious if the remaining thickness is decreased by controlling the corrosion process. In the experiment, H-shaped optical fibers with different losses were obtained by monitoring the loss change of PMFs in the corrosion process in real time, which means that H-shaped optical fibers have different remaining thicknesses. These fibers were coated with gold film and the transmission spectrum was tested for each. After the spectrum was obtained, the fiber was cut from the middle section, and the end face of the fiber was observed by a scanning electron microscope, which measured the remaining thickness. The spectra of different fibers and their corresponding remaining thicknesses are shown in Figure 4. They show that with the decrease in the remaining thickness, the depth of the resonant peak of the fiber increases significantly, but the wavelength position of the resonant peak does not change significantly.

**Figure 5:**
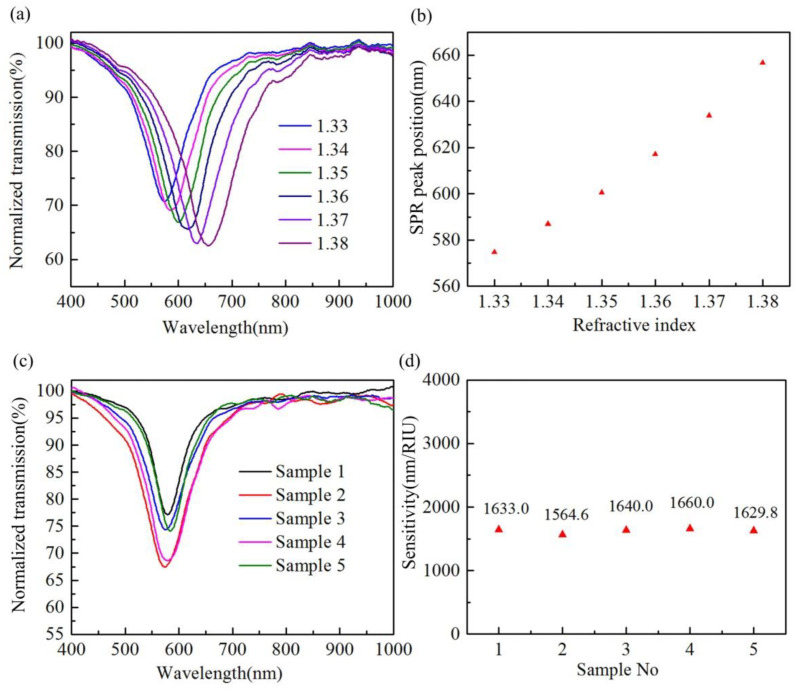
(**a**) Transmission spectrum of the H-shaped optical fiber (the length, remaining thickness and gold layer thickness are 1000 μm, 1 μm and 50 nm, respectively) under different external refractive indices; (**b**) variation in resonant peak wavelength with external refractive index; (**c**) transmission spectra of different fibers prepared in the same corrosion experiment; and (**d**) corresponding refractive index sensitivity of each fiber.

In order to probe the response characteristics of the proposed compact H-shaped optical fiber SPR sensor to the external refractive index, a commercial standard refractive index matching solution was used as the test solution. First, an H-shaped optical fiber with a length, remaining thickness, and gold layer thickness of 1000 μm, 1 μm, and 50 nm, respectively, was prepared, and then the fiber was tested with matching solutions with different refractive index values. Figure 5a shows the spectral evolution of the fiber when the refractive index increased from 1.33 to 1.38. It shows that as the refractive index increases, the SPR peak shows the same response characteristics as other optical fiber SPR sensors; that is, it drifts to the long wavelength direction. Figure 5b shows the change in resonant peak wavelength when the refractive index increases. When the external refractive index increased from 1.33 to 1.38, the resonant peak wavelength moved from 574.72 nm to 656.72 nm, a total wavelength shift of 82 nm, and the corresponding average refractive index is 1640 nm/RIU.

One advantage of the chemical etching method is that it can easily process multiple samples at the same time, and the characteristics of batch preparation are key to the large-scale application of the sensor. In order to verify the sensitivity consistency of the sensor, five PMF were corroded at the same time in a single corrosion experiment. After the corrosion, these fibers were coated and their spectra tested. Figure 5c shows that there are differences in the resonance peak intensities of different fibers. This may be mainly due to the slight difference in the remaining thickness after corrosion caused by the difference in the transverse structure of PMF in the samples, which results in different SPR excitation intensities of different fibers. The difference in resonant peak wavelength is mainly caused by the difference in gold film thickness between H-shaped optical fibers. Figure 3a shows that the gold film thickness has a significant impact on the position of the resonance peak of the H-shaped optical fiber. However, due to the coating technology, it is difficult to ensure that the gold film thickness between fibers is completely consistent. The refractive index responses of the five fibers were measured. Figure 5d shows that the refractive index sensitivity values of the fibers have good consistency. The average refractive index sensitivity of the five H-shaped optical fibers is 1625.48 nm/RIU and the maximum sensitivity error is 3.74%.

**Figure 6:**
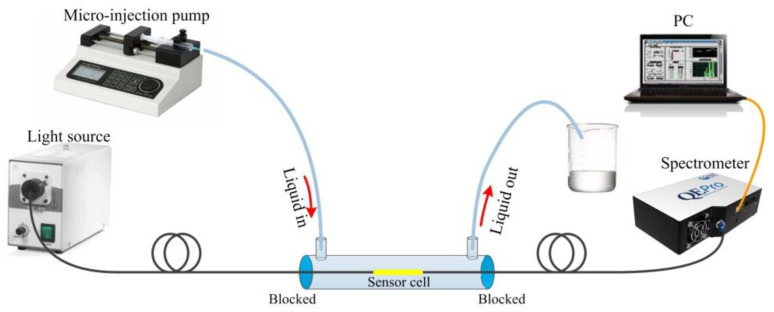
Schematic diagram of sensing test system for detecting human IgG.

The experimental setup of human IgG sensing is shown in Figure 6. The sensing area is sealed in a capillary, both ends of which are blocked by a UV curing adhesive. An inlet and an outlet channel are built at the two ends, respectively. The fiber sample is connected with a halogen lamp at one end and with a spectrometer (Ocean Optics, Largo, FL, USA) at the other end, and the transmission spectrum was recorded and displayed by a computer. In the sensing process, different solutions are injected into the capillary from the liquid inlet by a micro-injection pump. After flowing through the H-shaped optical fiber sensing area, the solution finally flows into the waste liquid bottle through the liquid outlet.

**Figure 7:**
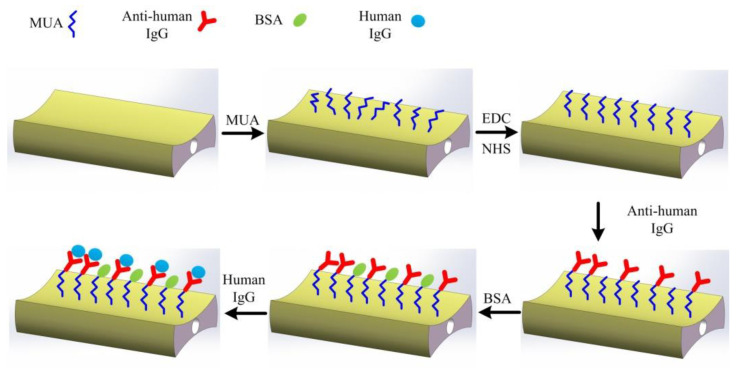
Schematic diagram of H-shaped optical fiber surface modification and human IgG detection process.

As shown in Figure 7, the surface modification of the H-shaped optical fiber and the human-IgG detection process is as follows:

(1) Clean the fiber with deionized water and ethanol to ensure that contaminants are removed from the fiber surface.
(2) 11-Mercaptoundecanoic acid (MUA) solution (50 mM) was injected into the capillary tube to completely soak the gold-coated H-shaped optical fiber at room temperature for 12 h. After that, the H-shaped optical fiber was washed with ethanol and PBS buffer. The sulfhydryl groups (-SH) of MUA combined with the gold film, forming solid Au–S bonds, and the carboxyl groups of MUA were exposed to the outside.
(3) An aqueous solution of a mixture of EDC/NHS (0.4 mM/0.1 mM) was injected into the capillary tube to completely soak the sensing area at room temperature for 30 min to ensure the carboxylic group of the thiolated surface was activated. Then, the H-shaped optical fiber was fully rinsed with PBS buffer.
(4) The goat anti-human IgG was dissolved in PBS at a concentration of 200 μg/mL and then the solution was injected into the capillary to flow across the sensing area. Two hours later, the H-shaped optical fiber was fully rinsed with a PBS buffer.
(5) Bovine Serum Albumin (BSA) solution (0.1 g/mL) was used to wash the fiber to block the residual unbound sites, and then the fiber was rinsed with PBS buffer.
(6) A surface-modified H-shaped optical fiber was used to monitor different concentrations of human IgG solutions. After each test, the bonds between human IgG and goat anti-human IgG were broken 10 min by 10 mM NaOH [28,29], and then the H-shaped optical fiber was rinsed with PBS buffer; subsequently, the test for the next concentration started.

**Figure 8:**
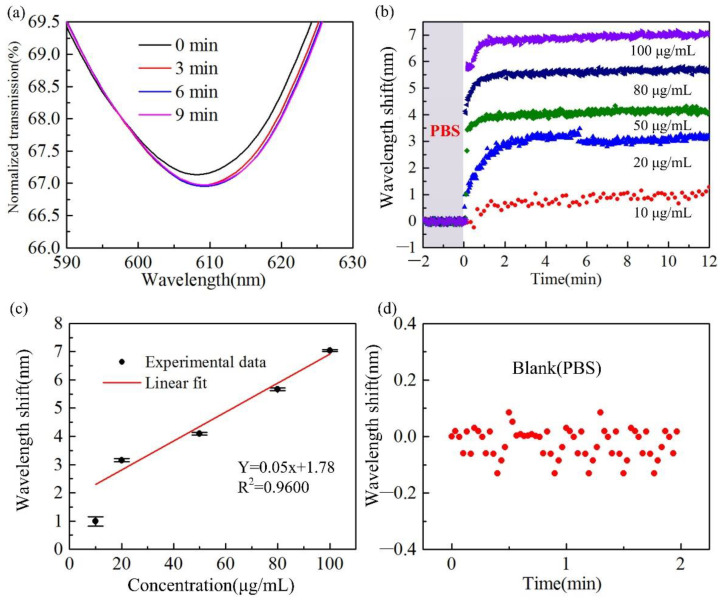
(**a**) The evolution of the H-shaped optical fiber spectrum with time when 10 μg/mL concentration human IgG is monitored. (**b**) Relative shift of resonant wavelength in human IgG test with different concentrations. (**c**) The resonant wavelength responses to the change of human IgG concentration. (**d**) The wavelength shifts of the sensor in PBS.

The changes in the transmission spectrum of the H-shaped optical fiber sensor in the process of detecting different concentrations of human IgG are shown in Figure 8. Before each test, the optical fiber was washed with PBS buffer, and the position of the resonant peak in PBS was used as a reference. When the solution to be tested flows across the sensing area, the transmission spectrum shifts toward longer wavelengths gradually. This is due to the surrounding refractive index increasing the fiber induced by the background solution and the combination of human IgG and goat anti-human IgG at the fiber surface. Figure 8a shows the change in the H-shaped optical fiber spectrum when testing 10 μg/mL concentration human IgG solution. It shows a spectrum shift to the long wavelength direction. Figure 8b shows the relative shift of the resonant peak wavelength with the increase in time when human IgG solutions with different concentrations are tested. This shows that the sensor takes longer to complete the specific binding of human IgG and goat anti-human IgG at low concentrations. When a high concentration human IgG solution enters the sensing area, the spectrum of the H-shaped optical fiber will shift greatly within a few seconds; then, due to the high concentration of human IgG, the spectral shift caused by specific binding can be completed in less than three minutes. The resonant wavelength responses to the change in human IgG concentration are represented in Figure 8c. The black dots represent the relationship between the resonant wavelength shift and the human IgG concentration, and the red solid line proves the calibration curve (R^2^ = 0.9600). The sensor sensitivity is calculated to be 0.05 nm/(μg/mL) according to the slopes of fitting lines. Figure 8d presents the wavelength shifts of the sensor in PBS 2 min, according to which the mean of wavelength shift (∆λ_blank_) and the standard deviation (σ) were determined to be 0.02 nm and 0.05 nm, respectively. The LOD was calculated as 3.4 μg/mL by the formula of *C_LOD_* = *f*^−1^(∆λ_blank_ + 3σ), described in previous research [30,31], where *f*^−1^ is the inverse of the fitting function.

**Figure 9:**
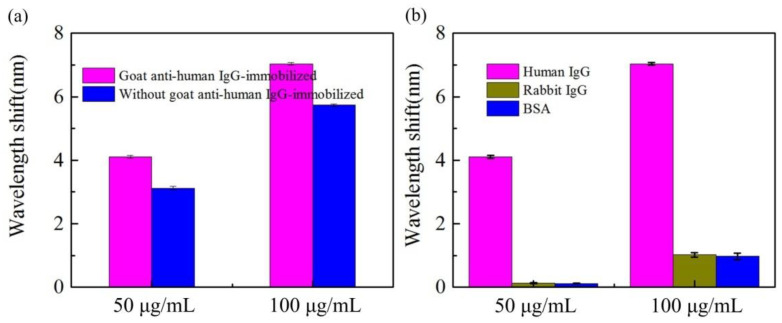
(**a**) Shifts in the resonance wavelength when human IgG is detected by the sensor (with and without immobilized goat anti-human IgG). (**b**) Shifts in the resonant wavelength measured with different antigens (human IgG, rabbit IgG, and BSA) based on the H-shaped optical fiber sensor.

In order to demonstrate the binding interaction between goat anti-human IgG and human IgG in the detection process, piranha solution was used to regenerate the gold film on the surface of optical fiber [28]. The same surface functionalization process with the goat anti-human IgG immobilized sensor was used to modify the surface of the gold film regenerated optical fiber, but the step of goat anti-human IgG immobilization was omitted, and this fiber without goat anti-human IgG immobilization was used as a reference for human IgG detection. As shown in Figure 9a, when the concentrations of human IgG solution to be detected are 50 and 100 μg/mL, respectively, the wavelength shifts of the goat anti-human IgG immobilized sensor are 4.10 and 7.04 nm, respectively, while the wavelength shift of the sensor without goat anti-human IgG immobilized is 3.12 and 5.74 nm, respectively, which indicates that there has binding interaction between goat anti-human IgG and human IgG during the human IgG detection process of the goat anti-human IgG immobilized sensor.

The specific detection for human IgG measurement is investigated by comparing the detection with rabbit IgG and BSA. Figure 9b presents the wavelength shifts when bathing the H-shaped optical fiber sensor in human IgG solutions, rabbit IgG solutions, and BSA solutions with 50 and 100 μg/mL concentrations for 12 min. The wavelength shifts are 4.10, 0.14, and 0.12 nm for human IgG, rabbit IgG, and BSA, with a concentration of 50 μg/mL, respectively. Lastly, the corresponding values for the 100 μg/mL solutions are 7.04, 1.02, and 0.98 nm. The scale values measured in the rabbit IgG and BSA solutions are relatively lower than the values in the human IgG solution, indicating that the surface-functionalized H-shaped optical fiber sensor can realize a selectivity detector for human IgG with a low cross-sensitivity to other agents.

## 4. Conclusions

Based on an H-shaped optical fiber, we demonstrate a compact fiber SPR biosensor. The chemical corrosion method is used to prepare the H-shaped optical fiber SPR sensors and the sensor prepared by this method has highly adjustable sensing unit length, good surface quality, and can prepare multiple samples at the same time. The effects of coating thickness, remaining thickness and H-shaped optical fiber length on the spectrum of the sensor were studied in detail. The results show that the best coating thickness is 50 nm, the optimal remaining thickness is 0 µm, and the length of the H-shaped optical fiber only needs to be 250 µm, which can realize obvious resonance peak. Finally, the proposed compact fiber SPR sensor was used to detect human IgG specifically. The results show that the sensor can detect human IgG with a concentration as low as 3.4 µg/mL.
